# PW01-012 – Canakinumab in patients with FMF

**DOI:** 10.1186/1546-0096-11-S1-A65

**Published:** 2013-11-08

**Authors:** S Ugurlu, E Seyahi, G Hatemi, A Hacioglu, H Ozdogan

**Affiliations:** 1 Department of Internal Medicine, Division of Rheumatology, Cerrahpasa Medical Faculty, University of Istanbul, Istanbul, Turkey

## Introduction

In a recent pilot study, it was reported that Canakinumab reduced the frequency of attacks in 9 patients with Familial Mediterranean Fever (FMF) resistant to colchicine with no apparent side effects [1].

## Objectives

Here, we present our experience with Canakinumab in FMF patients with insufficient response to colchicine.

## Methods

The charts of the patients with FMF who were on Canakinumab were evaluated retrospectively and the patients who had received 3 or more injections were asked to come to the clinic to assess the response and safety.

## Results

There were 19 patients with FMF (13 F/6 M) who were receiving canakinumab for various indications. Here we report 10 (6 F/4 M) who had at least 3 injections. Three patients had concomitant diseases such as psoriasis, ankylosing spondylitis and polyarteritis nodosa. The indications for canakinumab (150mg) were colchicine resistancy in 7 patients (>1 attack/month), amyloidosis in 2 and injection site reaction due to anakinra in one. The mean age of the patients was 31.8 ± 16.47 years, while the disease duration was 22.0 ± 9.98 years. The mean colchicine dose was 2.28 ± 0.36 mg / day. The median injection number with canakinumab was 4 (range 3-7). Although injections were planned to be monthly, patients received the drug with irregular intervals due to shortage of the drug. The duration of canakinumab use was 4 ± 1.2 months. Eight of the patients had no attacks after canakinumab, while in two patients attack frequency was reduced more than 50%. In two patients with amyloidosis, proteinuria was stable in one and increased from 1.7g/d to 4.7g/d in the other. Six of the patients who were complaining of severe myalgia, improved significantly after treatment. According to patient global assessment nine patients reported significant improvement while only one, reported no change.

Canakinumab was tolerated well in general. None of the patients had injection site reactions. Although, the patient with psoriasis reported a flare in psoriatic plaques, the treatment was not interrupted and psoriasis was controlled eventually by local applications.

## Conclusion

Canakinumab is effective in decreasing the frequency of attacks in colchicine-resistant FMF patients. In spite of the small number of patients, and short duration of follow-up, the side effect profile seems favorable. There is need for larger trials to further evaluate its efficacy on amyloidosis.

## Disclosure of interest

None declared
